# Causal association of circulating metabolites with diabetic retinopathy: a bidirectional Mendelian randomization analysis

**DOI:** 10.3389/fendo.2024.1359502

**Published:** 2024-05-10

**Authors:** Bo Li, Xu Zhao, Wanrun Xie, Zhenzhen Hong, Ye Cao, Yan Ding, Yi Zhang

**Affiliations:** ^1^ Department of Endocrinology, Quanzhou First Hospital, Affiliated to Fujian Medical University, Quanzhou, Fujian, China; ^2^ Hubei Key Laboratory of Embryonic Stem Cell Research, Biomedical Research Institute, Hubei University of Medicine, Shiyan, Hubei, China; ^3^ Emergency and Critical Care Center, Renmin Hospital, Hubei University of Medicine, Shiyan, Hubei, China; ^4^ Department of Cardiology, Fujian Provincial Hospital, Shengli Clinical Medical College, Fujian Medical University, Fuzhou, Fujian, China; ^5^ Department of Cardiology, Renmin Hospital, Hubei University of Medicine, Shiyan, Hubei, China

**Keywords:** diabetic retinopathy, metabolites, causal relationship, diabetes mellitus, MR analysis

## Abstract

**Introduction:**

The retina is a highly metabolically active tissue, and there is a lack of clarity about the relationship between metabolites and diabetic retinopathy (DR). This study used two-sample bidirectional Mendelian randomization (MR) analyses to identify causal relationships between metabolites and DR.

**Methods:**

Genetic variants were selected from the open-access Genome-Wide Association Studies (GWAS) summary database as proxies for the 1400 most recently published metabolites. MR analysis was performed to examine associations between these metabolite traits and DR. Single nucleotide polymorphism (SNP) data that were significantly associated with exposure were screened through association analysis. Validated instrumental variables (IVs) were obtained by removing SNPs with linkage disequilibrium (LD) and F-statistic values below 10. MR analyses were performed using the inverse variance weighted (IVW) method as the primary approach. The robustness of the results was verified by sensitivity analyses, including assessments of heterogeneity, horizontal pleiotropy, and the leave-one-out method.

**Results:**

In the IVW approach and in the primary analysis of several sensitivity analyses, genetically determined glycolithocholate sulfate levels, androstenediol (3 beta, 17 beta) monosulfate (1) levels, 1-stearoyl-2-arachidonoyl-GPE (18:0/20:4) levels, 1-oleoyl-2-arachidonoyl-GPE (18:1/20:4) levels, 1-oleoyl-2-linoleoyl-GPE (18:1/18:2) levels, X-26109 levels, N6-methyllysine levels, (N6,N6-dimethyllysine levels), and (N2-acetyl,N6,N6-dimethyllysine levels) were negatively associated with the risk of DR. 5-hydroxymethyl-2-furoylcarnitine levels and the glutamate-to-alanine ratio were positively associated with the risk of DR. No reverse causal association was found between DR and metabolites.

**Discussion:**

This MR study suggests that nine metabolites may have a protective effect in DR, while two metabolites may be associated with an increased risk of DR. However, further research is needed to confirm these findings. Supplementation with beneficial metabolites may reduce DR risk and could potentially be a novel therapeutic approach to DR treatment.

## Introduction

DR is one of the common complications of diabetes mellitus (DM), which seriously endangers human health and is the leading cause of vision loss and blindness (1). DM is a systemic metabolic disorder in which chronic hyperglycemic exposure causes widespread damage to neurons and vascular cells. Diabetic vision hazards primarily affect the retina (2). Structural and functional changes occur in retinal neurons and blood vessels, along with abnormalities in metabolic immunity, which further disrupt the blood-retinal barrier. Ultimately, these factors lead to neuronal damage. Currently, DR lacks effective early treatment and prevention measures. Exploring the pathogenesis and etiology of the disease is beneficial for early intervention.

The retina is a metabolically highly active tissue that requires the interaction of cells ranging from light-sensitive photoreceptors to neurons that transmit electrochemical signals to the brain (2). Currently, there is a lack of clarity about the relationship between metabolites and DR. Meta-analyses of DR studies have shown that blood pressure, serum total cholesterol, and glycosylated hemoglobin levels are associated with retinopathy. However, these factors account for only 9% of DR progression (3). Metabolomics is an emerging scientific field that has gained popularity in the biomedical community. It is used to diagnose diseases, understand disease mechanisms, and identify new drug targets. It has had a profound impact on unraveling the underlying causes of complex diseases, drug discovery, and precision medicine (4). Approximately 50% of phenotypic differences at metabolite level are attributed to genetic variation (5). This provides an opportunity to make causal inferences from metabolites to diseases. Alleles are randomly assigned at conception, and this randomization process helps eliminate the confounding effect of most risk factors, reducing confounding bias (6).

The Mendelian randomization approach is an epidemiological method that uses genetic variation as an instrumental variable to evaluate causality, while accounting for confounding factors. Genotyping can be a powerful tool for investigating causal relationships between exposure factors and outcomes. To investigate the mechanisms of DR, we hypothesized that there is a correlation between metabolite traits and DR. In our study, we used a two-sample bidirectional MR analysis to establish the causal relationship between metabolites and DR.

## Materials and methods

### Study design

**Figure 1:**
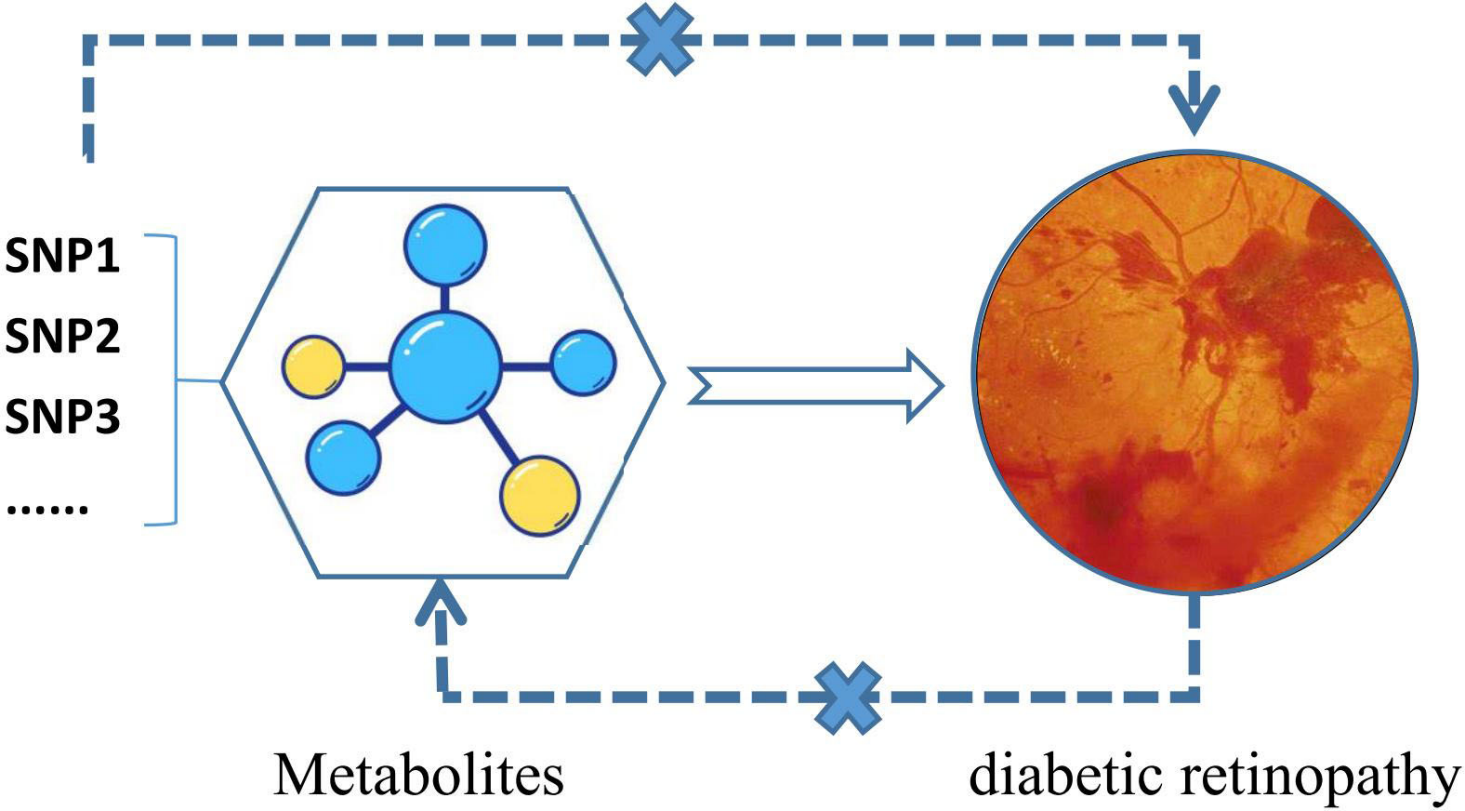
Flowchart of bidirectional Mendelian randomization analysis of metabolites and DR.

We assessed the causal relationship between 1400 metabolite traits and DR using a two-sample MR analysis. SNPs that were significantly associated with exposure were used as exposure proxies, while SNPs in linkage disequilibrium (LD) and weak IVs were excluded from MR analysis. Effective causal inference IVs must satisfy three essential assumptions: (1) IVs must have a direct relationship with the exposure variable; (2) IVs must be independent of potential confounding variables between exposure and outcome; and (3) IVs must not affect outcome through any other pathways than exposure. Ethical consent was previously obtained for GWAS primary studies in each country, and current analyses are based on publicly available summary-level data that do not require additional approval (**Figure 1**).

### GWAS data sources for DR

Summary GWAS data for the DR were obtained from the IEU Open GWAS project (https://gwas.mrcieu.ac.uk/). The study analyzed 190,594 European men and women (Ncase = 14,584, Ncontrol = 176,010) and examined 16,380,347 genetic variants.

### GWAS metabolites data source

Metabolite data were obtained from 8299 individuals in the Canadian Longitudinal Study of Aging (CLSA) cohort study. The GWAS summary study was performed by aggregating 1,091 blood metabolites and 309 metabolite ratios. 850 Blood metabolites were derived from known superpathways, while 241 metabolites were either unknown or only partially known. Metabolite-gene and gene expression information were integrated to identify genes with significant effects on traits and diseases. Genetic signatures of 109 metabolites and 48 metabolite ratios were ultimately identified as effector genes (6).

### Selection of IVs

In the analysis of metabolite-DR associations, we established a correlation threshold of 1×10^-5^ to identify SNPs that are significantly associated with metabolites. The r^2^ cutoff clustering was set to 0.001, and the clustering window was set to 10,000 kb to eliminate LD. F-statistics $\left(\text{F}=\frac{\text{N}-\text{K}-1}{\text{K}}\frac{R^{2}}{1-R^{2}}\right)$ were calculated for each IV (7), and any F value less than 10 was considered a weak IV and therefore excluded. When exposure and outcome were harmonized, palindromic SNPs with intermediate allele frequencies were removed. For DR and metabolite MR analysis, we set the correlation threshold at 5 × 10^-8^ to screen for SNPs significantly correlated with DR. We applied the same criteria to screen for IVs.

### Statistical analysis

To investigate the causal relationship between 1400 metabolite traits and DR, we used five methods (MR Egger, weighted median, IVW, simple mode, and weighted mode) from the TwoSampleMR package for analysis. The IVW method was used as the primary statistic, and sensitivity analyses were conducted using the weighted mode and weighted median methods. IVW, weighted median, and weighted mode were used to analyze and present the results. Heterogeneity was assessed using funnel plots and Cochran’s Q p values from IVW and MR-Egger tests, with *p* < 0.05 indicating heterogeneity. The horizontal pleiotropy of the data and the robustness of the results were assessed using MR-Egger intercepts. A *p*-value of less than 0.05 indicated that horizontal pleiotropy was excluded (8). Horizontal pleiotropy was also assessed using the MR-PRESSO global test, and peripheral SNPs were excluded by the MR-PRESSO outlier test (9). Finally, leave-one-out and single-SNP analyses were used to determine whether individual SNPs influenced primary causality (10).

In addition, the false positive rate increased due to the large number of exposure factors and multiple exposure phenotypes in GWAS derived from the same sample set. To address this, we applied the false discovery rate (FDR < 0.2) to correct MR results.

Ethical consent was obtained prior to the initial GWAS studies in each country, and the current analyses were based on publicly available summary data, which did not require further approval.

TwoSampleMR (version 0.5.6) in R (version 4.2.3) were used for MR analysis.

## Results

**Figure 2:**
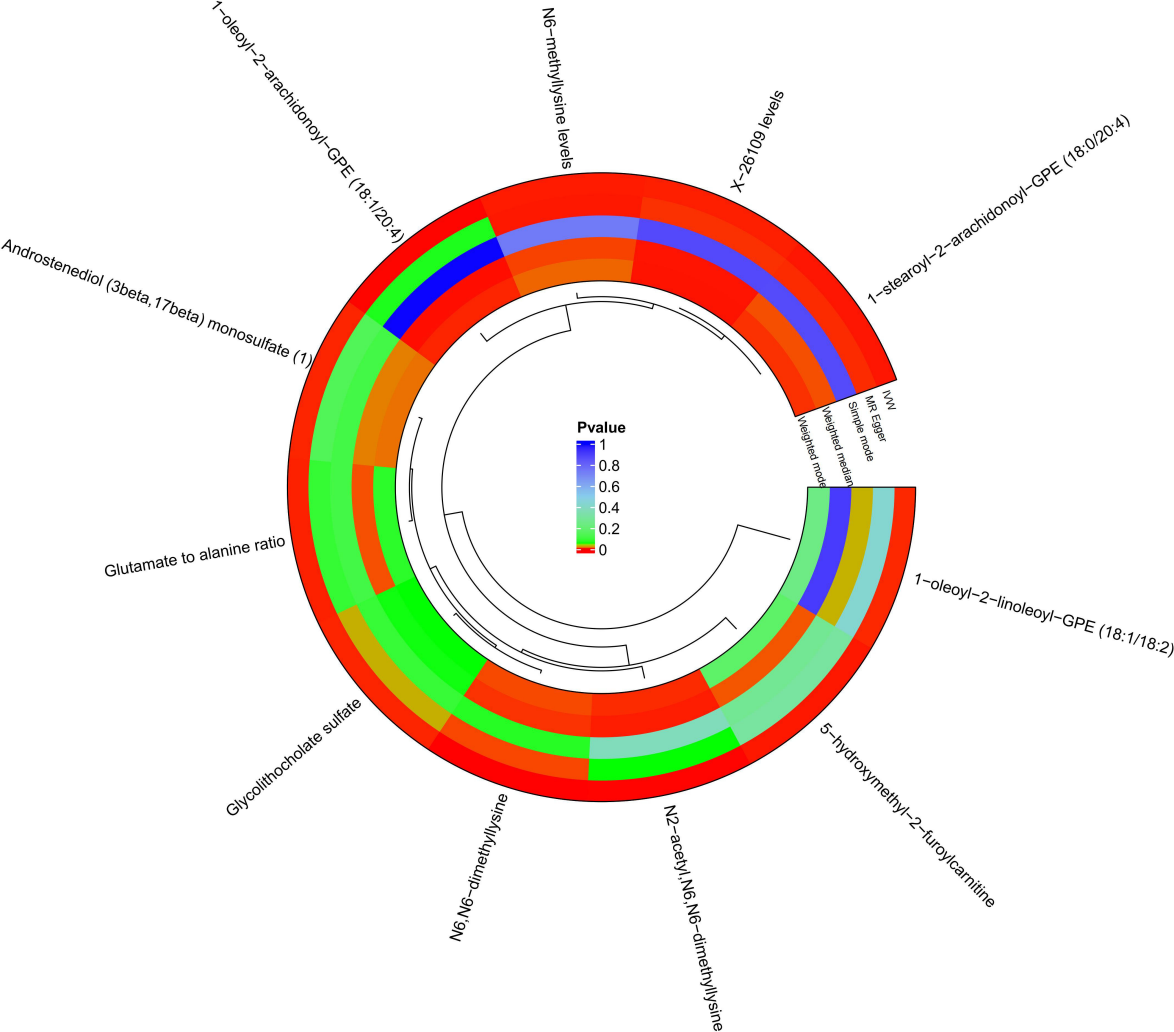
Circle plots of five MR analyses of 11 metabolites with significant causal effects with diabetic retinopathy. MR methods with p<0.05 are shown in red.

A total of 34,843 SNPs were screened as IVs for 1,400 metabolites, using IV screening criteria. The association results of 1400 metabolites with DR were obtained using five MR analysis methods, and 94 metabolites were found to be causally associated with DR according to IVW method. The MR results were filtered using the following criteria: *p* < 0.05 and FDR < 0.2 in the IVW method, consistency in the direction of β-value across five MR methods, and *p* > 0.05 to account for horizontal pleiotropy. Finally, 11 metabolites were found to be causally associated with DR (**Figure 2**; **Supplementary Figures S1, S2**). Reverse MR analysis did not reveal an association between DR and these 11 metabolites.

### Effects of metabolites on DR

**Figure 3:**
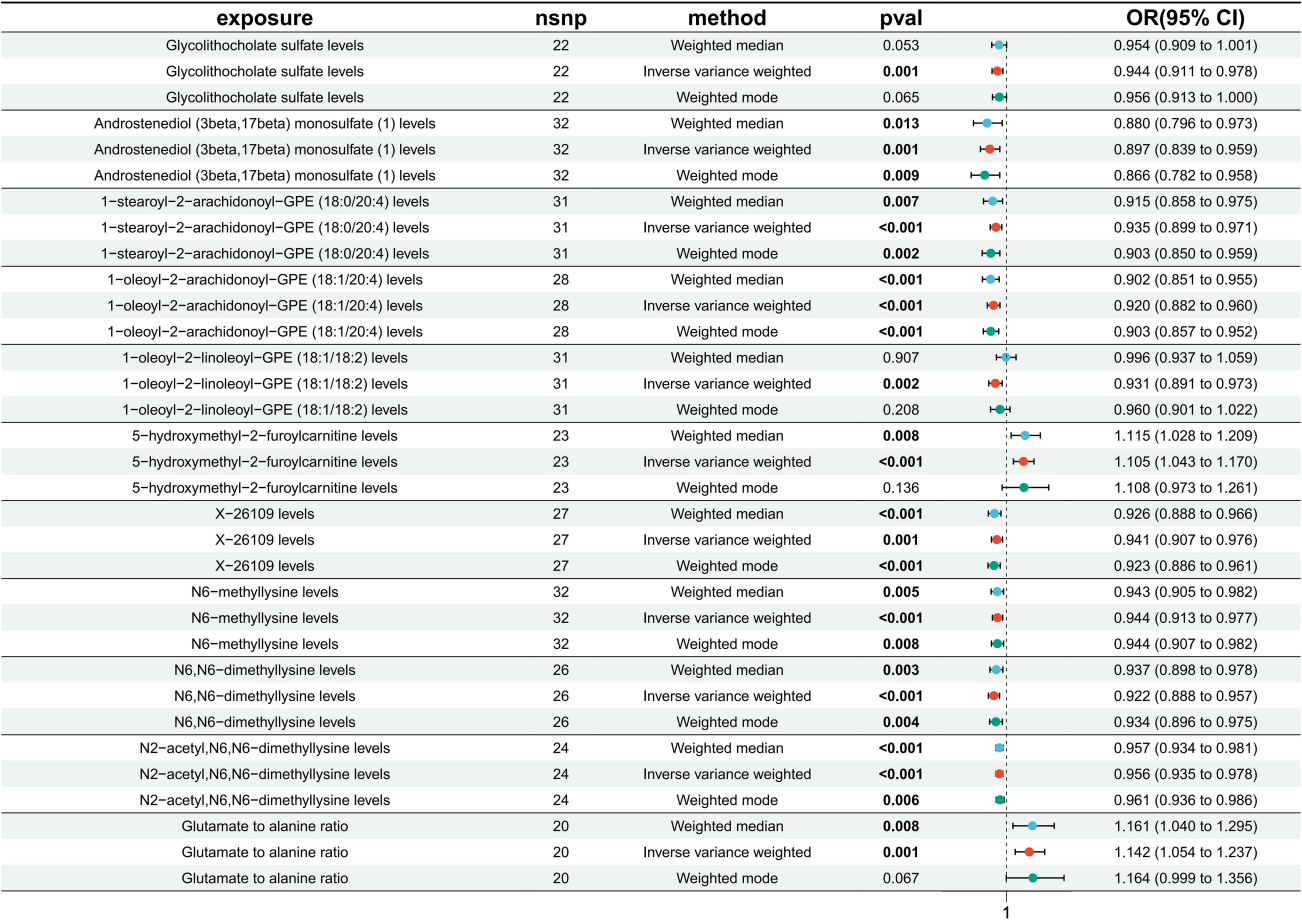
Results of MR analysis of the causal effects of 11 circulating metabolites on the risk of diabetic retinopathy (DR). Each line segment depicts the association between a specific metabolite and DR risk. A line segment to the right of 1 indicates a possible positive association, while a line segment to the left of 1 suggests a negative association. Finally, an intersection of this line segment with a vertical line at 1 indicates no association.

Glycolithocholate sulfate levels (IVW: β= -0.0578, OR = 0.944, 95%CI=0.911-0.987, *p* = 0.001), androstenediol (3 beta, 17 beta) monosulfate (1) levels (IVW: β= -0.109, OR = 0.897, 95%CI=0.839-0.959, *p* = 0.001), 1-stearoyl-2-arachidonoyl-GPE (18:0/20:4) levels (IVW: β= -0.067, OR = 0.935, 95%CI=0.899-0.971, *p* < 0.001), 1−oleoyl−2−arachidonoyl−GPE (18:1/20:4) levels (IVW: β= -0.084, OR = 0.920, 95%CI=0.882-0.960, *p* < 0.001), 1−oleoyl−2−linoleoyl−GPE (18:1/18:2) levels (IVW: β= -0.072, OR = 0.931, 95%CI=0.891-0.973, *p* = 0.002), X−26109 levels (IVW: β= -0.061, OR = 0.941, 95%CI=0.907-0.976, *p* = 0.001), N6−methyllysine levels (IVW: β= -0.057, OR = 0.944, 95%CI=0.913-0.977, *p* < 0.001), N6,N6−dimethyllysine levels (IVW: β= -0.081, OR = 0.922, 95%CI=0.888-0.957, *p* < 0.001), N2−acetyl,N6,N6−dimethyllysine levels (IVW: β= -0.045, OR = 0.956, 95%CI=0.935-0.978, *p* < 0.001) were negatively associated with the risk of DR. 5-hydroxymethyl-2-furoylcarnitine levels (IVW: β= 0.100, OR = 1.105, 95%CI=1.043-1.170, *p* < 0.001) and the glutamate-to-alanine ratio (IVW: β= 0.132, OR = 1.142, 95%CI=1.054-1.237, *p* = 0.001) were positively associated with the risk of DR (**Figure 3**).

### Pleiotropy and sensitivity analysis

Sensitivity and pleiotropy analyses validated the robustness of the IVW results. IVW and MR-Egger heterogeneity tests showed no heterogeneity in glycolithocholate sulfate levels, androstenediol (3 beta, 17 beta) monosulfate (1) levels, 1-stearoyl-2-arachidonoyl-GPE (18:0/20:4) levels, 1-oleoyl-2-arachidonoyl-GPE (18:1/20:4) levels, 1-oleoyl-2-linoleoyl-GPE (18:1/18:2) levels, X-26109 levels, N6-methyllysine levels, N6,N6-dimethyllysine levels, and N2-acetyl,N6,N6-dimethyllysine levels in MR analysis with DR (*p*>0.05) (**Table 1**; **Supplementary Figure S3**).

**Table 1:** Results of metabolite and DR heterogeneity and horizontal pleiotropy tests.

Heterogeneity				Pleiotropy		
ID	Exposure	MR Egger Q_p	IVW Q_p	Egger intercept	Egger P	MR-Presso p
GCST90199811	Glycolithocholate sulfate levels	0.253	0.304	-0.001	0.927	0.396
GCST90199872	Androstenediol (3beta,17beta) monosulfate (1) levels	0.196	0.227	-0.002	0.729	0.266
GCST90200045	1-stearoyl-2-arachidonoyl-GPE (18:0/20:4) levels	0.634	0.509	0.012	0.078	0.428
GCST90200079	1-oleoyl-2-arachidonoyl-GPE (18:1/20:4) levels	0.305	0.350	-0.002	0.794	0.396
GCST90200082	1-oleoyl-2-linoleoyl-GPE (18:1/18:2) levels	0.254	0.236	-0.008	0.252	0.135
GCST90200244	5-hydroxymethyl-2-furoylcarnitine levels	0.865	0.890	0.004	0.662	0.902
GCST90200671	X-26109 levels	0.276	0.228	0.008	0.171	0.286
GCST90200689	N6-methyllysine levels	0.918	0.816	0.011	0.057	0.862
GCST90200696	N6,N6-dimethyllysine levels	0.381	0.417	0.005	0.568	0.459
GCST90200697	N2-acetyl,N6,N6-dimethyllysine levels	0.851	0.818	-0.007	0.223	0.857
GCST90200946	Glutamate to alanine ratio	0.952	0.966	-0.004	0.732	0.967

Q_p, Cochran’s Q p-values.

The MR-Egger intercept and MR-Presso tests showed no evidence of horizontal pleiotropy (**Table 1**). The robustness and reliability of the causal relationship between metabolites and DR have been further confirmed using sensitivity analyses on leave-one-out (**Supplementary Figure S4**).

### Causal effects of DR onset on metabolites

**Figure 4:**
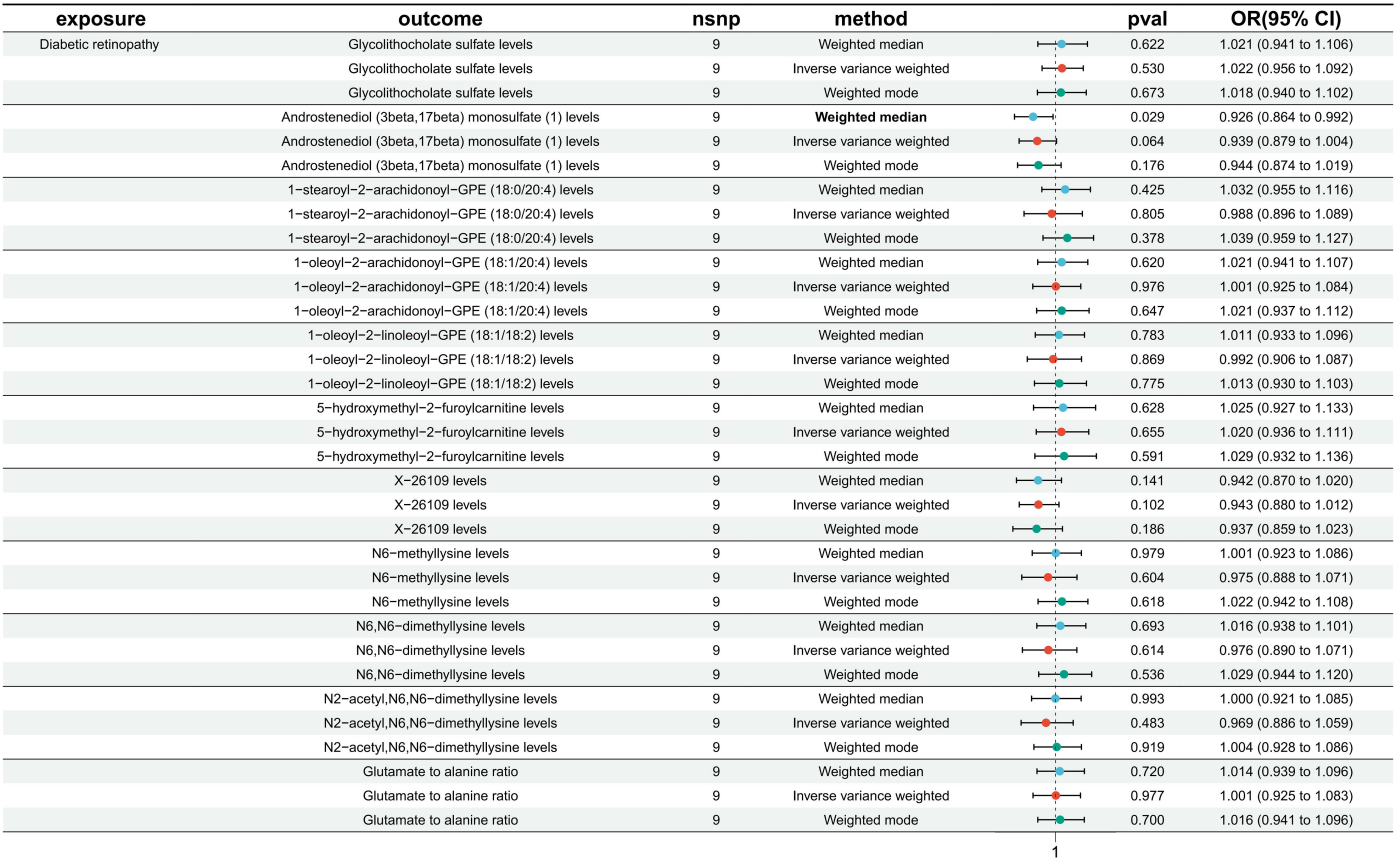
Causal effect analysis between DR and 11 metabolites. All lines intersect 1, indicating no reverse causal effect between them.

No reverse causal association was found between metabolites and DR (**Figure 4**).

### Effects of metabolites on T2DM

To estimate the causal effect of metabolites with diabetes, we performed MR analysis of 1400 circulating metabolites with T2DM (nCase: 38841,nControl: 451,248, nSNP: 24167560, Dataset:ebi-a-GCST90018926). 24 Metabolites showed a potential causal association (**Supplementary Figure S5**). Further Venn intersection analysis of DR and T2DM-related metabolites revealed that only 1-oleoyl-2-linoleoyl-GPE (18:1/18:2) levels had a negative phase causal effect with both DR and T2DM (**Supplementary Figure S6**).

## Discussion

DR is a common chronic complication of diabetes that primarily affects the retina of the eye. The retina, which is part of the central nervous system, is characterized by a high metabolism and a demand matched by a large supply of metabolites (11). Despite strict control of risk factors such as blood pressure and glucose that reduce DR risk, many diabetic patients continue to develop DR (12). Metabolic memory has been used to explain this phenomenon, whereby persistent epigenetic modifications induced by early exposure to hyperglycemia predispose individuals to diabetic complications even when good glycemic control is achieved (13). Metabolites are closely associated with disease onset and progression. Han et al. identified 311 differential metabolites by metabolite analysis of retinal tissues from diabetic and nondiabetic mice, and DR metabolites were significantly enriched in purine metabolic signals. Adenosine, guanine and inosine have higher sensitivity, specificity and accuracy for DR prediction (14), suggesting that metabolic phenotypes (metabolites) associated with disease states may be related to disease mechanisms and pathophysiology, and could be used in individualized medicine or public health (13). Metabolomic analysis of the vitreous suggested increased lactate and glucose abundance in value-added DR (PDR) and significant reductions in galactose-pure and ascorbic acid (15). Plasma or serum remains the biofluid of choice in metabolomics studies due to the invasive nature of vitreous sampling limiting the replicable and translational potential of the study (12). Analysis of plasma metabolomics from DR patients showed that plasma metabolites complemented traditional risk factors and facilitated risk stratification of patients in the early stages of DR, and that the plasma metabolic phenotype of DR is unique and not just a continuation of diabetic plasma metabolism (12), and that studying DR metabolomics is valuable for DR prevention and treatment and risk stratification.

DR is a chronic metabolic disease, and dysregulation of retinal metabolism is a key factor in DR pathogenesis (16). Circulating metabolomics analyses have significant translational value in the study of DR mechanisms, risk stratification, and the development of new therapeutic measures (12). Changes in the metabolome represent the interaction of genetic and environmental factors and provide information complementary to genomic, transcriptomic or proteomic data (12). A recent 12-year follow-up on metabolites and DR in Finnish men with type 2 diabetes showed that 17 metabolites were significantly associated with incident DR, among which N-lactoyl isoleucine, N-lactoyl valine, N-lactoyl tyrosine, N-lactoyl phenylalanine, N-(2- furoyl) glycine, N,N,N-trimethyl-5-aminovalerate,carboxylic acid maleate, 3-hydroxypyridine sulfate, 4-vinylphenol sulfate, 4-ethylcatechol sulfate, dimethyl sulfoneand 5-hydroxylysine were associated with an increased risk of DR, and citrulline, myristoleate (14:1n5), palmitoleate (16. 1n7), Sphingomyelin (d18:2/24:2), and 5-dodecenoate (12:1n7) with a decreased risk of DR (17). Another nested case-control metabolomics study found 11 metabolites (1,5-Anhydroglucitol, 1,5-Gluconolactone, 2-Deoxyribonic acid, 3,4-Dihydroxybutyric acid, Erythritol, Gluconic acid, Lactose/cellobiose, Maltose/trehalose, Mannose, Ribose, Urea) was associated with DR, and correction for metabolic risk factors and renal function did not significantly affect the results (12). Our results showed that the following metabolites were negatively associated with the risk of DR: glycolithocholate sulfate levels, androstenediol (3 beta, 17 beta) monosulfate (1) levels, 1-stearoyl-2-arachidonoyl-GPE (18:0/20:4) levels, 1-oleoyl-2-arachidonoyl-GPE (18:1/20:4) levels, 1-oleoyl-2-linoleoyl-GPE (18:1/18:2) levels, X-26109 levels, N6-methyllysine levels, N6,N6-dimethyllysine levels, and N2-acetyl,N6,N6-dimethyllysine levels. On the other hand, 5-hydroxymethyl-2-furoylcarnitine levels and glutamate-to-alanine ratios were positively associated with DR risk. No reverse causal effect was found between metabolites and DR. MR analyses from UK Biobank data showed no association of circulating metabolites with fasting glucose and 2-hour glucose, and 19 circulating metabolites were associated with a lower risk of type 2 diabetes (18). However, these metabolites did not intersect with those associated with DR risk in our analysis. This may be related to the different levels of refinement and classificationbolites.

Metabolomics is the omics discipline closest to phenotype and can be used not only to explore important biomarkers of disease, but also to identify metabolites that may alter the phenotype of a cell or organism (19). The combination of metabolomics and systems biology information can test endogenous metabolites for phenotype-altering functions, and there is growing evidence that metabolites regulate a variety of biological processes (19). Metabolites are important bridges between genes, proteins and phenotypes. They are downstream of gene transcription and translation, and also support and regulate molecules in the microenvironment where gene expression and environmental exposure are regulated (20). Not only does it respond to the health status of the organism, but it can also be an effective window to regulate the state of the organism. Because metabolites are often readily available, especially for multifactorial diseases, they are considered a very powerful instrument with great potential for clinical translation (20). Although there is an increasing number of metabolomics studies in the eye, metabolomics research is still in its early stages and there are still many questions to be solved. Our study provides some theoretical support for DR mechanism research, prevention and risk stratification.

MR analysis methods. Results were robust and unaffected by horizontal pleiotropy or other confounding factors. Our studies have some limitations. The study is based on an individual of European ancestry, so conclusions cannot be extrapolated to the entire population. MR studies of DR in GWAS cohorts from other different ancestry groups (Africans, African Americans, Europeans, Hispanics, and Asians) will provide insights into how different genetic compositions resulting from inter-ethnic diversity and different environments lead to varying degrees of causal effects of circulating metabolites on DR development. Secondly, despite sensitivity analyses, horizontal pleiotropy could not be completely eliminated. Finally, using a lower threshold to assess the causal relationship between metabolite traits and DR may increase the number of false positives, but it allows for a more comprehensive assessment.

In addition, MR analysis predicts trends associated with DR risk and does not reflect metabolite disorders at different stages of disease, so more comprehensive, multimetric assessment strategies are needed to analyze different aspects of metabolite change.

## Conclusions

We confirmed a causal relationship between a variety of metabolites and DR through bidirectional MR analysis, highlighting the complex features of the interactions between the metabolites and DR.

Our study significantly reduced the effects of reverse causality, confounding factors that are difficult to exclude, and other factors, providing new ideas for further exploration of the biological mechanisms of DR and some guidance for early treatment and prevention of DR.

## Data availability statement

The original contributions presented in the study are included in the article/**Supplementary Material**. Further inquiries can be directed to the corresponding authors.

## Author contributions

BL: Conceptualization, Software, Writing – original draft. XZ: Data curation, Investigation, Writing – review & editing. WX: Supervision, Writing – original draft. ZH: Project administration, Validation, Writing – original draft. YC: Resources, Writing – review & editing. YD: Writing – review & editing. YZ: Visualization, Writing – review & editing.
