# Supplementary figures and images for: Causal association of circulating metabolites with diabetic retinopathy: a bidirectional Mendelian randomization analysis
Source: Front Endocrinol (Lausanne). 2024 May 10;15:1359502. doi: 10.3389/fendo.2024.1359502 (PMC11116606; doi:10.3389/fendo.2024.1359502)

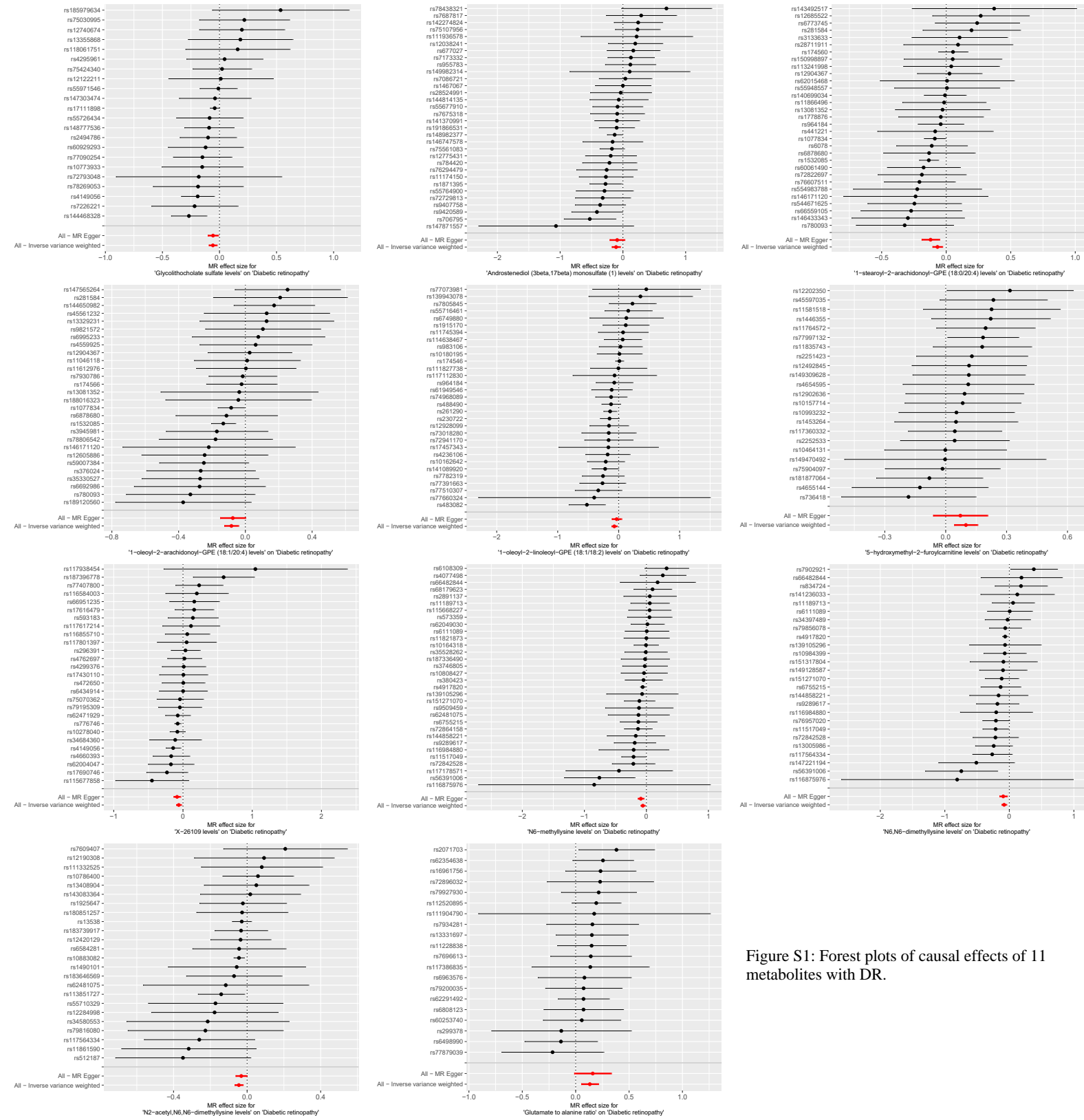

Figure S1: Forest plots of causal effects of 11 metabolites with DR.

Supplement: Supplementary file 1 [file Image_1.pdf]

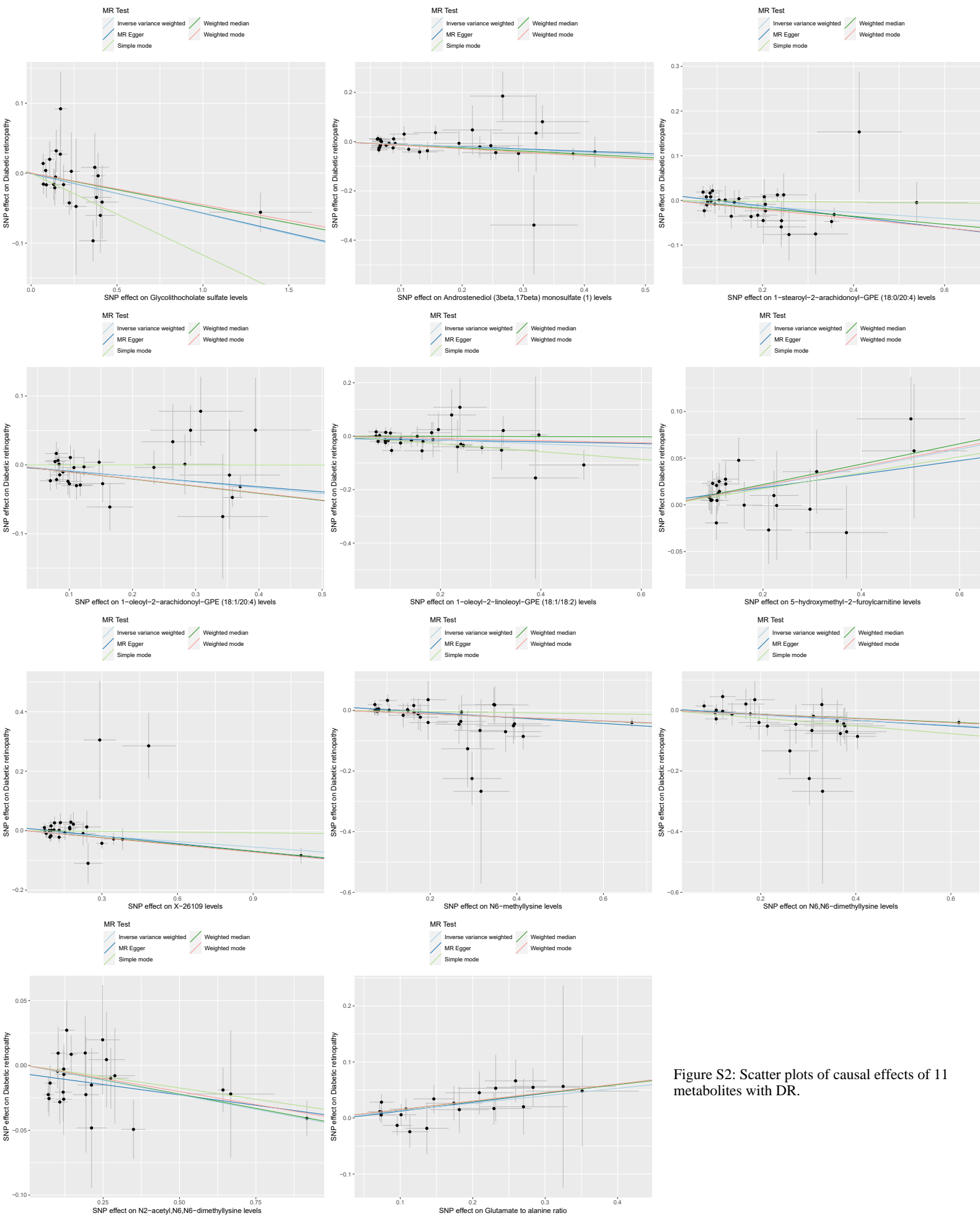

Figure S2: Scatter plots of causal effects of 11 metabolites with DR.

Supplement: Supplementary file 2 [file Image_2.pdf]

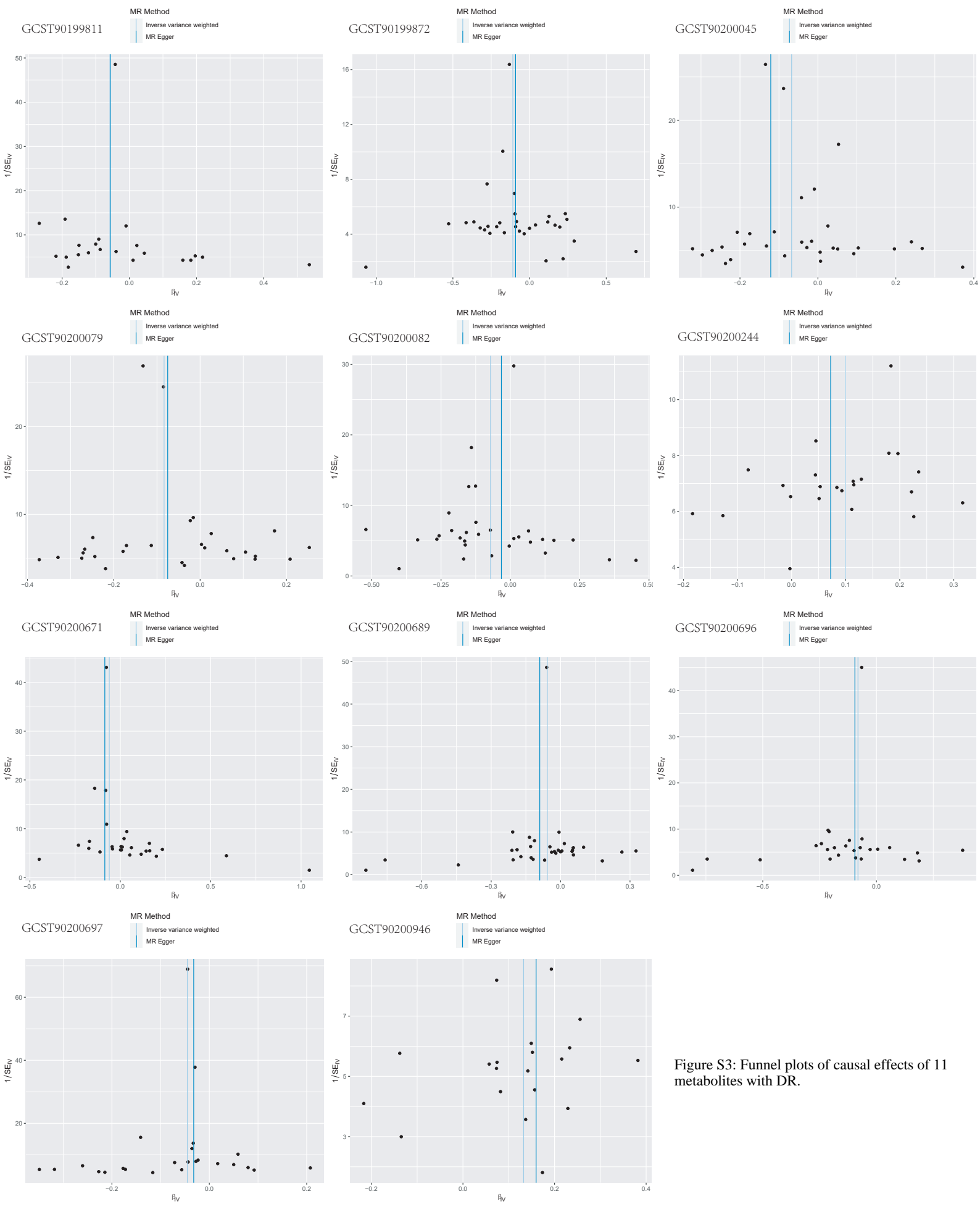

Figure S3: Funnel plots of causal effects of 11 metabolites with DR.

Supplement: Supplementary file 3 [file Image_3.pdf]

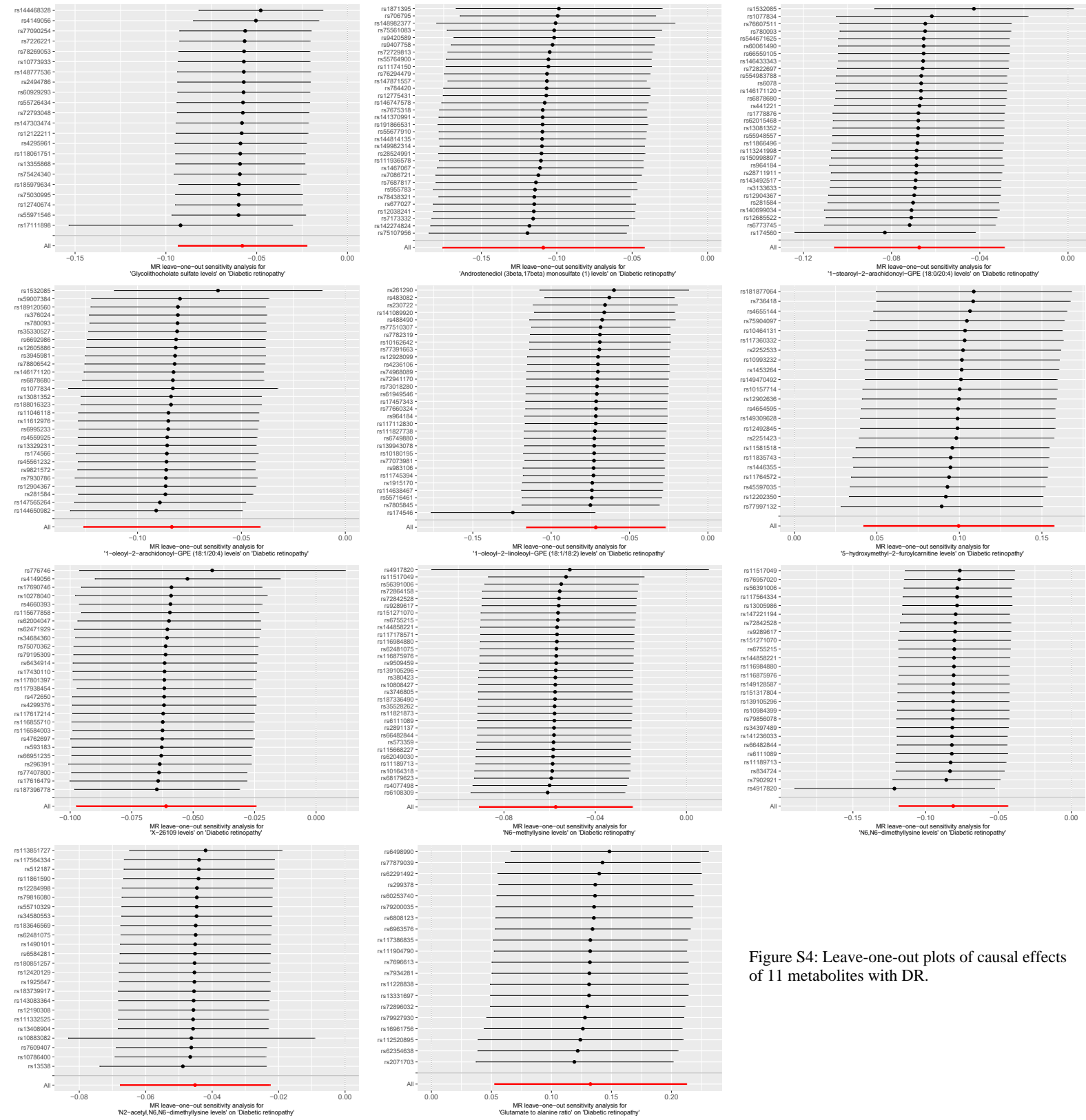

Figure S4: Leave-one-out plots of causal effects of 11 metabolites with DR.

Supplement: Supplementary file 4 [file Image_4.pdf]

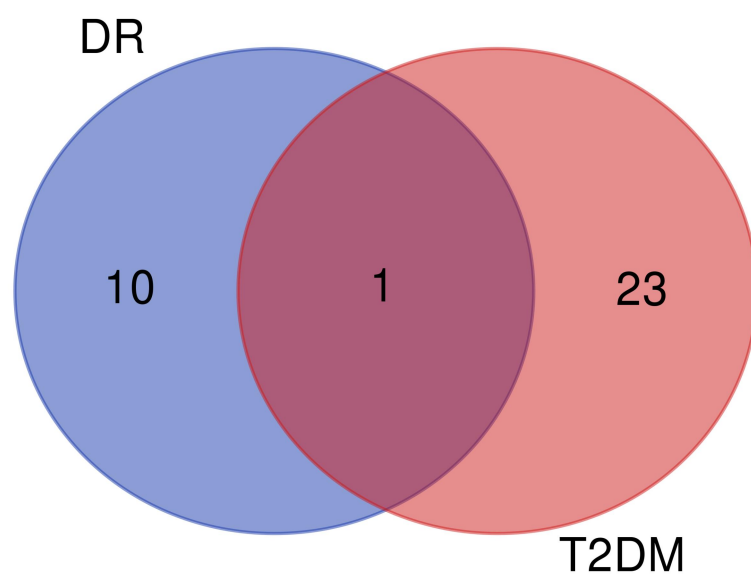

Figure S6: Venn diagram of the intersection of 11 DR metabolites and 24 T2DM metabolites

Supplement: Supplementary file 6 [file Image_6.pdf]
