# Supplementary material for: Causal association of circulating metabolites with diabetic retinopathy: a bidirectional Mendelian randomization analysis
Source: Front Endocrinol (Lausanne). 2024 May 10;15:1359502. doi: 10.3389/fendo.2024.1359502 (PMC11116606; doi:10.3389/fendo.2024.1359502)
Supplement: Supplementary file 5 [file Image_5.pdf]

exposure	nsnp	method	pval	OR(95% CI)
4−methyl−2−oxopentanoate levels	12	Weighted median	**0.012**	1.100 (1.021 to 1.185)
4−methyl−2−oxopentanoate levels	12	Inverse variance weighted	**0.002**	1.089 (1.031 to 1.151)
4−methyl−2−oxopentanoate levels	12	Weighted mode	0.133	1.096 (0.981 to 1.226)
N−acetylaspartate (naa) levels	21	Weighted median	0.067	0.955 (0.908 to 1.003)
N−acetylaspartate (naa) levels	21	Inverse variance weighted	**0.001**	0.946 (0.915 to 0.979)
N−acetylaspartate (naa) levels	21	Weighted mode	0.108	0.954 (0.903 to 1.008)
Cysteine−glutathione disulfide levels	27	Weighted median	**0.047**	0.961 (0.924 to 0.999)
Cysteine−glutathione disulfide levels	27	Inverse variance weighted	**<0.001**	0.956 (0.931 to 0.981)
Cysteine−glutathione disulfide levels	27	Weighted mode	0.077	0.963 (0.924 to 1.002)
1−linoleoyl−GPE (18:2) levels	33	Weighted median	**<0.001**	0.910 (0.876 to 0.946)
1−linoleoyl−GPE (18:2) levels	33	Inverse variance weighted	**<0.001**	0.930 (0.899 to 0.962)
1−linoleoyl−GPE (18:2) levels	33	Weighted mode	**<0.001**	0.910 (0.874 to 0.948)
3−(3−hydroxyphenyl)propionate levels	21	Weighted median	**0.047**	1.058 (1.001 to 1.119)
3−(3−hydroxyphenyl)propionate levels	21	Inverse variance weighted	**0.004**	1.061 (1.020 to 1.104)
3−(3−hydroxyphenyl)propionate levels	21	Weighted mode	0.155	1.051 (0.984 to 1.123)
Vanillactate levels	24	Weighted median	0.305	1.033 (0.971 to 1.099)
Vanillactate levels	24	Inverse variance weighted	**0.003**	1.094 (1.030 to 1.162)
Vanillactate levels	24	Weighted mode	0.408	1.031 (0.960 to 1.108)
Sphingomyelin (d18:1/22:1, d18:2/22:0, d16:1/24:1) levels	22	Weighted median	**0.038**	1.074 (1.004 to 1.148)
Sphingomyelin (d18:1/22:1, d18:2/22:0, d16:1/24:1) levels	22	Inverse variance weighted	**<0.001**	1.095 (1.038 to 1.154)
Sphingomyelin (d18:1/22:1, d18:2/22:0, d16:1/24:1) levels	22	Weighted mode	0.109	1.087 (0.986 to 1.198)
1−palmitoyl−2−dihomo−linolenoyl−GPC (16:0/20:3n3 or 6) levels	28	Weighted median	**<0.001**	0.917 (0.887 to 0.949)
1−palmitoyl−2−dihomo−linolenoyl−GPC (16:0/20:3n3 or 6) levels	28	Inverse variance weighted	**<0.001**	0.945 (0.917 to 0.974)
1−palmitoyl−2−dihomo−linolenoyl−GPC (16:0/20:3n3 or 6) levels	28	Weighted mode	**<0.001**	0.921 (0.888 to 0.955)
1,2−dilinoleoyl−GPE (18:2/18:2) levels	28	Weighted median	0.299	0.977 (0.934 to 1.021)
1,2−dilinoleoyl−GPE (18:2/18:2) levels	28	Inverse variance weighted	**<0.001**	0.942 (0.917 to 0.968)
1,2−dilinoleoyl−GPE (18:2/18:2) levels	28	Weighted mode	0.607	0.981 (0.912 to 1.055)
1−oleoyl−2−linoleoyl−GPE (18:1/18:2) levels	32	Weighted median	**<0.001**	0.948 (0.921 to 0.976)
1−oleoyl−2−linoleoyl−GPE (18:1/18:2) levels	32	Inverse variance weighted	**0.003**	0.951 (0.920 to 0.984)
1−oleoyl−2−linoleoyl−GPE (18:1/18:2) levels	32	Weighted mode	**<0.001**	0.947 (0.924 to 0.971)
Ceramide (d18:1/17:0, d17:1/18:0) levels	24	Weighted median	0.052	1.039 (1.000 to 1.080)
Ceramide (d18:1/17:0, d17:1/18:0) levels	24	Inverse variance weighted	**<0.001**	1.053 (1.022 to 1.085)
Ceramide (d18:1/17:0, d17:1/18:0) levels	24	Weighted mode	**0.007**	1.055 (1.018 to 1.094)
Octadecadienedioate (C18:2−DC) levels	33	Weighted median	**0.018**	0.972 (0.950 to 0.995)
Octadecadienedioate (C18:2−DC) levels	33	Inverse variance weighted	**<0.001**	0.968 (0.951 to 0.986)
Octadecadienedioate (C18:2−DC) levels	33	Weighted mode	**0.008**	0.971 (0.951 to 0.991)
11beta−hydroxyetiocholanolone glucuronide levels	24	Weighted median	**0.007**	1.076 (1.020 to 1.135)
11beta−hydroxyetiocholanolone glucuronide levels	24	Inverse variance weighted	**0.002**	1.064 (1.022 to 1.107)
11beta−hydroxyetiocholanolone glucuronide levels	24	Weighted mode	0.145	1.078 (0.978 to 1.190)
Palmitoyl−sphingosine−phosphoethanolamine (d18:1/16:0) levels	29	Weighted median	0.144	1.041 (0.986 to 1.099)
Palmitoyl−sphingosine−phosphoethanolamine (d18:1/16:0) levels	29	Inverse variance weighted	**0.003**	1.086 (1.029 to 1.146)
Palmitoyl−sphingosine−phosphoethanolamine (d18:1/16:0) levels	29	Weighted mode	0.506	1.028 (0.949 to 1.113)
Branched chain 14:0 dicarboxylic acid levels	20	Weighted median	**0.048**	0.930 (0.865 to 0.999)
Branched chain 14:0 dicarboxylic acid levels	20	Inverse variance weighted	**0.001**	0.906 (0.853 to 0.962)
Branched chain 14:0 dicarboxylic acid levels	20	Weighted mode	0.437	0.957 (0.860 to 1.066)
Gamma−glutamylhistidine levels	24	Weighted median	0.091	0.958 (0.912 to 1.007)
Gamma−glutamylhistidine levels	24	Inverse variance weighted	**0.002**	0.950 (0.921 to 0.981)
Gamma−glutamylhistidine levels	24	Weighted mode	0.121	0.961 (0.916 to 1.009)
X−16397 levels	18	Weighted median	**0.037**	0.940 (0.888 to 0.996)
X−16397 levels	18	Inverse variance weighted	**0.002**	0.936 (0.897 to 0.976)
X−16397 levels	18	Weighted mode	0.091	0.925 (0.849 to 1.007)
X−21736 levels	33	Weighted median	0.231	1.032 (0.980 to 1.086)
X−21736 levels	33	Inverse variance weighted	**0.004**	1.053 (1.016 to 1.090)
X−21736 levels	33	Weighted mode	0.334	1.036 (0.965 to 1.112)
X−21829 levels	30	Weighted median	**0.008**	1.070 (1.018 to 1.125)
X−21829 levels	30	Inverse variance weighted	**<0.001**	1.074 (1.036 to 1.112)
X−21829 levels	30	Weighted mode	0.051	1.073 (1.003 to 1.149)
Alpha−ketoglutarate to glutamate ratio	32	Weighted median	0.118	0.956 (0.904 to 1.011)
Alpha−ketoglutarate to glutamate ratio	32	Inverse variance weighted	**0.001**	0.934 (0.896 to 0.974)
Alpha−ketoglutarate to glutamate ratio	32	Weighted mode	0.778	0.987 (0.899 to 1.083)
Phosphate to mannose ratio	26	Weighted median	**0.009**	0.913 (0.853 to 0.977)
Phosphate to mannose ratio	26	Inverse variance weighted	**<0.001**	0.870 (0.818 to 0.926)
Phosphate to mannose ratio	26	Weighted mode	0.580	0.945 (0.777 to 1.151)
Phenylalanine to tyrosine ratio	20	Weighted median	0.280	1.034 (0.973 to 1.099)
Phenylalanine to tyrosine ratio	20	Inverse variance weighted	**0.001**	1.070 (1.026 to 1.115)
Phenylalanine to tyrosine ratio	20	Weighted mode	0.697	1.021 (0.922 to 1.129)
Adenosine 5'−diphosphate (ADP) to N−acetylneuraminate ratio	16	Weighted median	**0.007**	1.075 (1.020 to 1.133)
Adenosine 5'−diphosphate (ADP) to N−acetylneuraminate ratio	16	Inverse variance weighted	**<0.001**	1.071 (1.032 to 1.111)
Adenosine 5'−diphosphate (ADP) to N−acetylneuraminate ratio	16	Weighted mode	0.059	1.102 (1.004 to 1.210)
Arachidonate (20:4n6) to caffeine ratio	23	Weighted median	0.526	1.017 (0.966 to 1.069)
Arachidonate (20:4n6) to caffeine ratio	23	Inverse variance weighted	**0.004**	1.065 (1.020 to 1.111)
Arachidonate (20:4n6) to caffeine ratio	23	Weighted mode	0.666	1.013 (0.955 to 1.075)

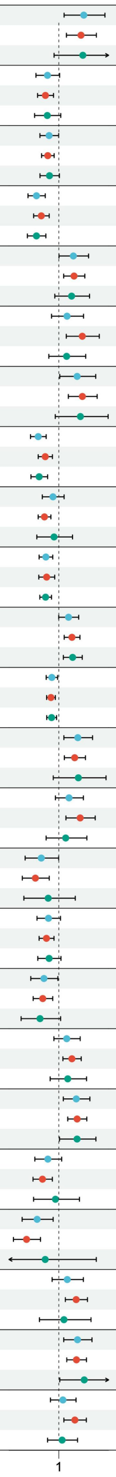

Figure S5: Results of MR analysis of the causal effects of 24 circulating metabolites on the risk of type 2 diabetes mellitus (T2DM). Each line segment depicts the association between a specific metabolite and T2DM risk. A line segment to the right of 1 indicates a possible positive association, while a line segment to the left of 1 suggests a negative association. Finally, an intersection of this line segment with a vertical line at 1 indicates no association.
